# A global dataset of biochar application effects on crop yield, soil properties, and greenhouse gas emissions

**DOI:** 10.1038/s41597-023-02867-9

**Published:** 2024-01-09

**Authors:** Xin Li, Dong Wu, Xue Liu, Yaping Huang, Andong Cai, Hu Xu, Jiwei Ran, Jing Xiao, Wenju Zhang

**Affiliations:** 1 State Key Laboratory of Efficient Utilization of Arid and Semi-arid Arable Land in Northern China, Key Laboratory of Arable Land Quality Monitoring and Evaluation, Ministry of Agriculture and Rural Affairs/Institute of Agricultural Resources and Regional Planning, Chinese Academy of Agricultural Sciences, Beijing, 100081 China; 2 TERRA Teaching and Research Centre, Gembloux AgroBio Tech, University of Liège, 5030 Gembloux, Belgium; 3 Key Laboratory of Agricultural Environment, Ministry of Agriculture and Rural Affairs, Institute of Environment and Sustainable Development in Agriculture, Chinese Academy of Agricultural Sciences, Beijing, 100081 China; 4 Key Laboratory of Plant Nutrition and the Agri-environment in Northwest China, Ministry of Agriculture, College Natural of Resources and Environment, Northwest A & F University, Yangling, 712100 Shaanxi China

**Keywords:** Phenology, Carbon cycle, Environmental impact

## Abstract

Biochar application is widely studied to mitigate the threats of soil degradation to food security and climate change. However, there are big variations in the effects of biochar application on crops, soils, and the atmosphere during crop production. This study provides a global dataset of biochar application effects on crop yield, soil properties, and greenhouse emissions. The dataset is extracted and integrated from 367 peer-reviewed studies with 891 independent field, laboratory, and incubation experiments across 37 countries. This dataset includes 21 variables before and after biochar application (including soil properties, crop yield, greenhouse gas emissions, etc.) of 2438 items, focusing on two main biochar application types: biochar application alone and combined with fertilizers. Background information on climate conditions, initial soil properties, management practices, and characteristics of biochar sources and production is also contained in the dataset. This dataset facilitates a comprehensive understanding of the impact of biochar application, supports the utilization of agricultural wastes for biochar production, and assists researchers in refining experimental protocols for further studies.

## Background & Summary

Sustainable agricultural production and climate change are two major challenges for agriculture. Biochar application has been highly recommended as a potential agricultural management practice to tackle the conflict between crop productivity and greenhouse gas emissions^1,2^. Biochar is a stable carbon-rich byproduct produced by the pyrolysis of biomass, such as agricultural and forestry waste, in the absence of oxygen^3^. With high carbon content, abundant pore structure, large specific surface area, and stable physicochemical properties, it has the potential to improve soil health and promote carbon sequestration^4^. Biochar incorporation can increase crop yield by ameliorating soil physical structure, improving nutrient availability, and enhancing microbial activities^5,6^. Besides, biochar application also has a profound impact on soil organic carbon decomposition and nitrogen transformation, which are mediated by microbial communities^3,7^.

There is big variability and high uncertainty in the effects of biochar application on crop yield, soil properties, and greenhouse gas emissions^8,9^. Previous meta-analysis studies suggest that biochar addition improved crop yield by 5–51%^8,10^, showing huge variations among studies. Soil organic carbon (SOC) under biochar application is 12–102% higher than that without biochar, according to previous meta-analysis studies^11,12^, showing the sequestration of soil carbon. Research also showed that biochar application also had positive effects on decreasing nitrate-nitrogen leaching and retaining ammonium-nitrogen, thereby altering the migration dynamics of soil nitrogen^13,14^. For greenhouse gas emissions, most studies demonstrate the potential of biochar application on CH_4_ and N_2_O emissions reduction^15–17^. Nevertheless, several studies illustrated there were no significant effects on greenhouse gas emissions^18^. Furthermore, a big variation lies in the effect of biochar application on greenhouse gas emissions from previous studies, ranging from 9–72%^19,20^ and 14–60%^20,21^ for the reduction of CH_4_ and N_2_O emissions, respectively. This could be the consequence of different management practices adopted under biochar application conditions^22^. Greenhouse gas fluxes may be influenced interactively by the co-application of biochar and fertilizers, such as chemical fertilizers and manure^23,24^. Therefore, it is necessary to distinguish between the effect of biochar alone and the interaction effect of biochar and fertilizers to minimize the uncertainty of the biochar application effect.

Initial soil properties, environmental conditions, biochar source, characteristics, and application rate are the main factors influencing the impacts of biochar on crop yield, soil properties, and greenhouse gas emissions. Climate conditions are widely recognized as a major factor, as crop growth requires both temperature and precipitation^7,25,26^. Additionally, differences in biochar characteristics and initial soil properties could significantly affect crop growth, carbon decomposition, and nitrogen transformation by regulating the degradation and biochemical cycling of biochar. For instance, previous research has demonstrated that the sources and pyrolysis temperatures of biochar greatly influence its properties^3^, which resulted in significant differences in the effect of biochar application^27–29^. Therefore, a comprehensive understanding of biochar derived from different sources and pyrolysis temperatures can help improve the utilization efficiency of agricultural wastes. Furthermore, there is an urgent need to quantify these factors on the effect size of biochar application on crop production, soil quality, and greenhouse gas emissions to evaluate the benefits and impacts of biochar application in agroecosystems.

**Fig. 1:**
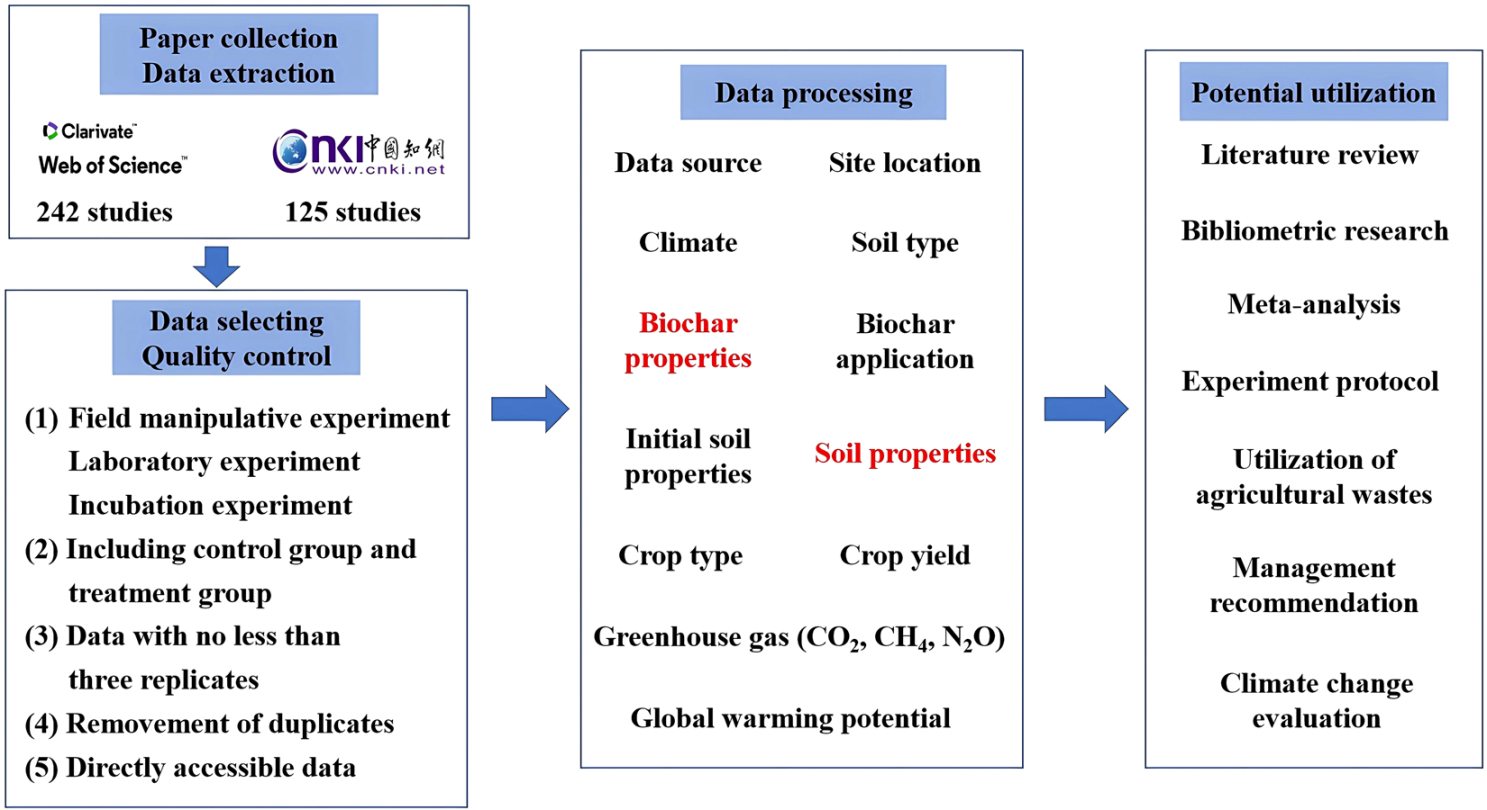
Data processing approach for data collection, screening, integrating, and the potential utilization of the dataset.

Here, we collected a dataset from peer-reviewed studies that compared crop yield, soil properties, and greenhouse gas emissions under three conditions: no biochar, biochar applied alone, and biochar combined fertilizers. Only peer-reviewed studies meeting specific criteria were digitized and integrated into our dataset. In this dataset, 891 independent experiments derived globally from 367 studies were synthesized to examine the response of biochar application. This dataset consists of 21 variables under control and biochar application treatments, including soil properties, crop yield, and greenhouse gas emissions. Information from the original studies on data sources, climate conditions, biochar properties, management practices, and initial soil properties is also included in this dataset. We intend to continue expanding the dataset and are open to contributions of additional data. Newly published data could be added to this open-source dataset for future real-time updates. The dataset can be performed for literature reviews and bibliometric research to explore research progress and frontiers. Furthermore, meta-analyses based on this dataset can examine the effect size of biochar on crop yield, soil properties, and greenhouse emissions from a global perspective. This dataset has significance in recommending appropriate management practices for farmers, stakeholders, and scientists, enabling them to develop sustainable agricultural strategies and production policies. Moreover, it could serve as a reference for biochar production with agricultural waste utilization (Fig. 1).

## Methods

### Data collection

To establish a comprehensive dataset on the effect of biochar on crop yield, soil properties, and greenhouse gas emissions in the global agricultural ecosystem, we implemented strict criteria to obtain closely relevant data from the Web of Science (https://www.webofscience.com/wos/alldb/basic-search), Google Scholar (https://www.scholar.google.com), and China National Knowledge Infrastructure (http://www.cnki.net). We used the keywords “biochar” and “crop productivity” or “crop yield” or “grain yield” or the keywords “biochar” and “soil properties” or “soil indicators” to conduct our research. All the selected studies and experimental data adhered to the following requirements: (a) Manipulative field, laboratory, and incubation experiments were conducted. Targeted experiments included a control (with no biochar addition) group and a treatment group (with biochar addition), with each group having no less than three plots as replicates; (b) No biochar was applied before or during the targeted experiments in the control group. For treatment groups, studies were obliged to specify whether biochar was applied alone (B) or combined with fertilizers (BF); (c) Data on soil properties, crop yield, and greenhouse gas emissions under B and BF was recorded in the studies; (d) Experimental data could be obtained directly from figures, tables, or text (Fig. 1). If necessary, GetData Graph Digitizer 2.24 was used to extract data from the figures.

**Fig. 2:**
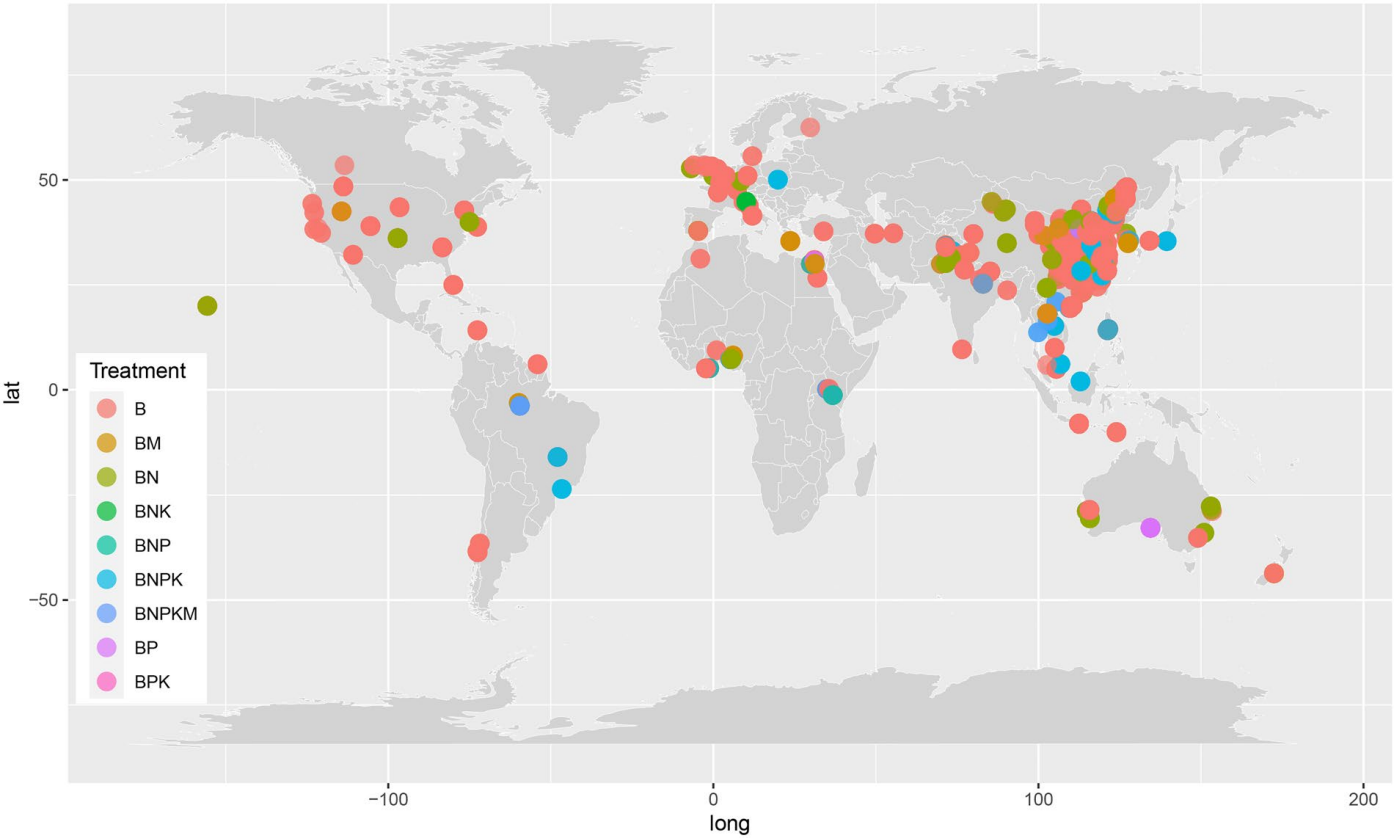
The spatial distribution of sites included in the dataset of biochar application effects. B, biochar application; BM, biochar application plus manure; BN, biochar application plus nitrogen fertilizer; BNK, biochar application plus nitrogen and potassium fertilizers; BNP, biochar application plus nitrogen and phosphorus fertilizers; BNPK, biochar application plus nitrogen, phosphorus, and potassium fertilizers; BNPKM, biochar application plus nitrogen, phosphorus, potassium fertilizers, and manure; BP, biochar application plus phosphorus fertilizer; BPK, biochar application plus phosphorus and potassium fertilizers.

A total of 2438 paired items under biochar addition experiments from 367 published studies were collected after searching and selection. The spatial distribution of the targeted sites is shown in Fig. 2. These studies consisted of 1686 B and 752 BF treatments. Among them, 799 items provided data with SOC contents, 1984 with crop yield, and 542 with global warming potential (GWP) determinations. In addition to SOC, soil properties such as pH, total and available nitrogen and phosphorus, base cations, cation exchange capacity (CEC), and microbial biomass were also extracted from the selected studies. Besides, the dataset includes information on greenhouse gas intensity (GHGI), as well as CO_2_, CH_4_, and N_2_O emissions. Considering that biochar properties were significant influencing factors, we obtained and incorporated data on biochar type, application rate, pH, pyrolysis temperature, ash content, carbon content, and nitrogen content into the dataset. Furthermore, the dataset provides information on the first author, publication year, site location (country, latitude, and longitude), and climate variables (mean annual temperature (MAT) and precipitation (MAP)).

### Data processing

**Fig. 3:**
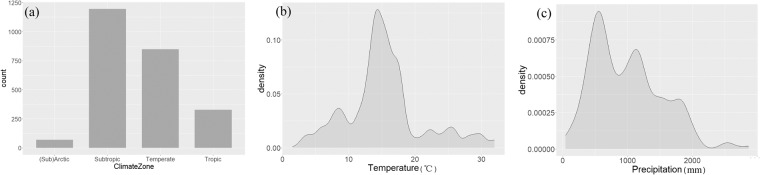
Distribution of samples included in the dataset of biochar application in different climate zones (**a**), mean annual temperature (**b**), and mean annual precipitation (**c**).

The climate zones of sites were supplemented in the dataset according to the latitude and longitude coordinates if no relevant information is provided in the studies. The climate zones were grouped into tropic (23.5 °S to 23.5 °N), subtropic (23.5 to 35 °S and °N), temperate (35 to 50 °S and °N), and (sub)arctic (>50 °S and °N) zones. Climate conditions, biochar properties, and initial soil properties were directly extracted from the targeted literature. The dataset covered a wide geographic range, with latitudes ranging from −43.65° to 62.50° and longitudes ranging from −155.69° to 172.46°. MAT varied from 1.5 °C to 32.0 °C, and MAP ranged from 45 mm to 2870 mm (Fig. 3). There were also wide variations in biochar application rate, biochar properties, and initial soil properties among different studies (Table 1). Soil texture was classified into sandy soil, loamy soil, and clayey soil based on the soil classification system of the U.S. Department of Agriculture. Soil pH was measured by different methods in our dataset. If soil pH was measured by the method of CaCl_2_ solution, the following equation was used to obtain the value of soil pH (H_2_O):

1 $$pH\left({H}_{2}O\right)=1.65+0.86\times pH\left(CaC{l}_{2}\right)$$

**Table 1:** Summary of site climate conditions, biochar properties, and soil properties in biochar application and biochar application combined with fertilizer treatments. SD indicates the standard deviation. *N* represents the number of data items.

Indexes	Unit	Biochar application			Biochar application combined with fertilizers		
		*N*	Mean (SD)	Range	*N*	Mean (SD)	Range
Site longitude	°	1686	87.48 (62.79)	−155.69–172.44	752	83.61 (59.61)	−155.69–172.46
Site latitude	°	1686	31.14 (15.45)	−43.64–62.50	752	25.46 (19.10)	−43.65–52.80
Wetness index		955	39.79 (17.29)	1.57–116.36	404	37.70 (15.58)	1.57–70.67
Biochar application rate	t ha^-1^	1686	27.42 (50.27)	0.04–900.00	752	18.72 (17.08)	0.05–112.50
Biochar pH		1451	9.22 (1.18)	4.15–13.00	663	9.06 (1.26)	5.70–13.00
Biochar carbon content	g kg^-1^	1329	529.26 (180.00)	4.97–981.80	655	544.62 (174.80)	4.97–876.70
Biochar nitrogen content	g kg^-1^	1368	9.12 (6.72)	0.30–80.00	668	9.25 (6.99)	0.30–68.00
Biochar phosphorus content	g kg^-1^	827	7.04 (12.45)	0.02–76.60	307	7.62 (12.29)	0.03–64.00
Initial soil organic carbon	g kg^-1^	1304	13.80 (9.62)	1.26–81.20	606	13.35 (7.12)	1.30–48.00
Initial total nitrogen	g kg^-1^	1100	1.29 (0.84)	0.01–10.22	549	1.38 (0.74)	0.01–4.00
Initial olsen phosphorus	mg kg^-1^	590	23.88(34.70)	0.04–470.58	268	30.96 (71.97)	0.09–570.00
Initial pH		1396	6.61 (1.36)	3.70–9.34	645	6.30 (1.29)	3.76–8.81
Initial bulk density	g cm^-3^	601	1.30 (0.17)	0.66–1.67	249	1.30 (0.15)	0.90–1.91
Initial cation exchange capacity	cmol kg^-1^	442	13.81 (10.46)	0.71–51.70	234	14.91 (10.87)	1.60–45.60

The wetness index was a combined measure of climate conditions, which was calculated using MAT and MAP as the following equation:

2 $${\rm{Wetness}}\;{\rm{index}}={\rm{MAP}}/({\rm{MAT}}+10)$$

The global warming potential (GWP) is an indicator of the total impact of greenhouse gases on the global greenhouse effect^30^. Based on the data on greenhouse gas (i.e., CO_2_, CH_4_, and N_2_O) emissions, the GWP was estimated using the following equation:

3 $$GWP=25\times {R}_{C{H}_{4}}+298\times {R}_{{N}_{2}O}+{R}_{C{O}_{2}}$$

where ${{\rm{R}}}_{{{\rm{co}}}_{2}}$, ${{\rm{R}}}_{{{\rm{CH}}}_{4}}$, and ${{\rm{R}}}_{{{\rm{CH}}}_{4}}$ are the soil CO_2_, CH_4_, and N_2_O emissions reported in the targeted literature (kg ha^−1^) respectively.

The greenhouse gas emission intensity (GHGI) is an indicator for a comprehensive evaluation of the greenhouse effect, which coordinates environmental benefits with crop yields^30^. It was calculated as follows:

4 $$GHGI=GWP/Yield$$

where GWP and Yield are global warming potential (kg ha^−1^) and crop yield (t ha^−1^) respectively.

Data of mean, number of replications, and standard deviations (SD) were compiled from the publications when possible. If standard error (SE) and coefficient of variation (CV) were reported in the study, SD was calculated as follows:

5 $$SD=SE\times \sqrt{n}$$

6 $$SD=CV\times mean$$

where n is the number of replications.

For the data without SD, SE, or CV, SD was calculated from the mean by the variance coefficient of all the datasets^31^.

## Data Records

All data and R code could be downloaded according to the FAIR principles^32^. The dataset is provided in spreadsheet format with the title “BiocharDS_V1.0”. This data file includes 2439 rows and 209 columns, containing data from 367 studies (The detailed references were listed in the sheet “Reference” and are matched with the data). Each column corresponds to specific information on study details, site location, climate conditions, initial soil properties, biochar properties, management information, soil properties, crop yield, and greenhouse gas emissions. Each row includes as many comparisons of data variables as possible between treatment and control. The explanations and units of the data are marked in the worksheet “Explanation and unit”. It should be noted that all the units of each property have been converted to the same unit even though units of the same property might differ. There are nine treatment groups: (1) biochar application alone (B); (2) biochar application plus manure (BM); (3) biochar application plus nitrogen fertilizer (BN); (4) biochar application plus nitrogen and potassium fertilizers (BNK); (5) biochar application plus nitrogen and phosphorus fertilizers (BNP); (6) biochar application plus nitrogen, phosphorus, and potassium fertilizers (BNPK); (7) biochar application plus nitrogen, phosphorus, potassium fertilizers, and manure (BNPKM); (8) biochar application plus phosphorus fertilizer (BP); (9) biochar application plus phosphorus and potassium fertilizers (BPK). All the treatments except B are collectively referred to as the BF group. The whole dataset is constructed of 891 independent experiments, including 700 under B and 314 under BF. “BiocharDS_V1.0.csv” is a simplified version of “BiocharDS_V1.0.xlsx” that only contains target information. There is another Rscript named “Biochar. R” that includes codes to generate the location of the sites and check the distribution of key information in the dataset. The “BiocharDS_V1.0.csv” file can be used to generate the figures.

## Technical Validation

The authenticity of the data to the original source was checked before data extraction. Duplicate studies were removed during selection. Each study was read at least twice, with particular attention given to the sections involving data (materials and methods, tables, figures, and supplementary materials). Data extracted from the tables or figures were cross-checked with the original files to ensure accurate digitization. Quality control was performed after data extraction to check the availability of the data. The formats of each column (numerical or string) were checked to correct any mistyping in the numerical columns.

After data extraction, we checked the distribution of all the variables. Outliers and/or extreme points were manually checked for potential errors by reviewing the data and units in the studies. The response ratio (RR) and natural log-transformed response ratio (Ln (RR)) of each variable of soil properties, crop yield, and greenhouse gas emissions were calculated. RR and Ln (RR) were calculated as formulas (7) and (8) respectively.

7 $$RR={M}_{t}/{M}_{c}$$

8 $$Ln\left(RR\right)=ln\left(\frac{{M}_{t}}{{M}_{c}}\right)=ln{M}_{t}-ln{M}_{c}$$

where Mt and Mc are the mean under the biochar application and control, respectively.

**Fig. 4:**
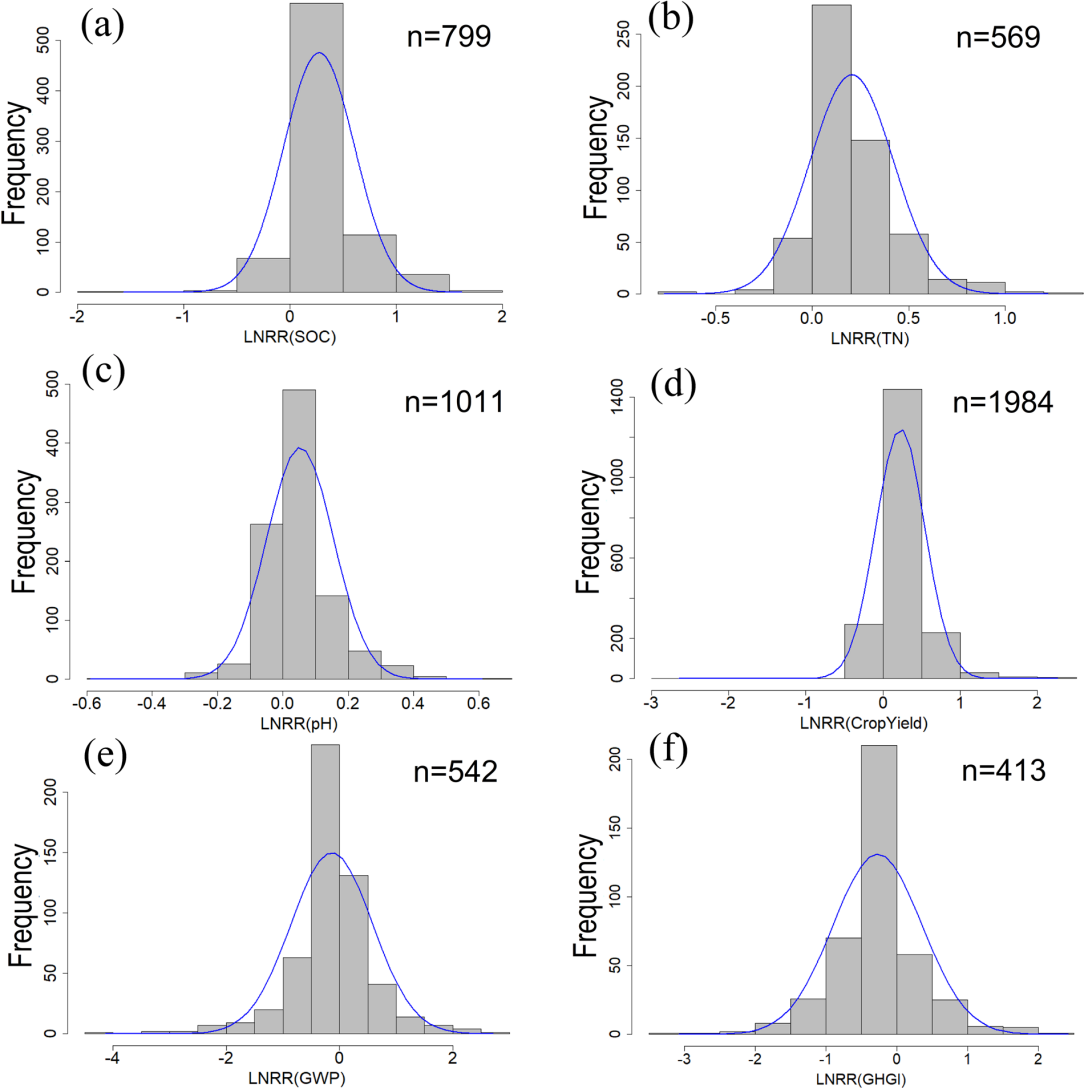
Frequency distribution of the natural log-transformed response ratio of biochar application on soil organic carbon (SOC) (**a**), total nitrogen (TN) (**b**), pH (**c**), crop yield (**d**), global warming potential (GWP) (**e**), and greenhouse gas emission intensity (GHGI) (**f**) in the dataset. The blue curve is a Gaussian distribution fitted to frequency data, and *P* < 0.01 suits the distribution.

The frequency distributions of Ln (RR) for some key variables were plotted to verify the normality of the data. The results showed the effects of biochar on SOC, TN, pH, crop yield, GWP, and GHGI varied greatly among experimental sites and displayed normal distributions (Fig. 4). For other variables, we compared the range of Ln (RR) of variables with other meta-analysis studies to ensure data availability^7–11,33–35^. Additionally, extreme values of Ln (RR) were validated from the original studies.

## Usage Notes

In the dataset of biochar application effects on crop yield, soil properties, and greenhouse gas emissions (BiocharDS_V1.0), the variables and units in each column are the same. Please note that the treatment group is classified not only based on the application of fertilizers and biochar but also according to the control treatment (e.g., the treatment under biochar application plus nitrogen, phosphorus, and potassium fertilizers is also B treatment if the control is under nitrogen, phosphorus, and potassium fertilizers). Soil sampling depths in the dataset are different between studies, and 90.48% of the items represent surface soil (soil depth ≤20 cm). This dataset is valuable for conducting literature reviews, bibliometric research, and meta-analyses of biochar application in agroecosystems. The inclusion of different biochar sources and properties in the dataset can provide a reference for improving the efficiency of re-utilizing agricultural waste resources. Moreover, it can serve as a reference for the evaluation of management practice selection for sustainable agricultural production and climate change mitigation. For any inquiries regarding code understanding or data usage, users can contact the corresponding author.

## Data Availability

Data visualization was conducted using R (version 3.5.1). The Rscript “BiocharDS_V1.0. R” includes codes to generate the location of the sites (Fig. 2) and check the distribution of key information in the dataset (Figs 3, 4). All the code and data used are available.
